# Supplementary material for: Mediterranean Diet and Healthy Eating in Subjects with Prediabetes from the Mollerussa Prospective Observational Cohort Study
Source: Nutrients. 2021 Jan 16;13(1):252. doi: 10.3390/nu13010252 (PMC7830064; doi:10.3390/nu13010252)
Supplement: Supplementary file 1 [file nutrients-13-00252-s001.pdf]

Supplementary Table S1. Daily food intake of the study groups.

Food Groups (g/day) [1]	Normal Glucose Tolerance (n = 319)	Prediabetes (n = 216)	p [2]
Dairy products	319.0 (191.0)	322.0 (212.0)	0.875
Eggs	21.5 (13.8)	23.3 (26.7)	0.690
White meat	34.3 (18.0)	35.0 (18.4)	0.860
Red meat	58.8 (34.2)	56.3 (27.0)	0.686
Processed meat	35.3 (25.7)	33.6 (24.9)	0.761
Meat	128.0 (48.1)	125.0 (40.6)	0.686
Lean fish	30.8 (22.6)	29.0 (19.8)	0.686
Fatty fish	27.7 (22.5)	28.4 (48.1)	0.873
Seafood	12.2 (10.8)	11.9 (10.0)	0.863
Total Fish	70.6 (37.3)	69.1 (56.1)	0.863
Vegetables	217.0 (104.0)	229.0 (103.0)	0.576
Fruits	239.0 (154.0)	221.0 (155.0)	0.576
Fruits and vegetables	456.0 (209.0)	450.0 (206.0)	0.863
Nuts	7.8 (9.6)	8.2 (8.7)	0.860
Legumes	21.9 (19.4)	22.8 (20.5)	0.860
Cereals and pasta	73.5 (43.1)	65.7 (38.3)	0.162
Potatoes	49.4 (30.0)	61.0 (66.0)	0.162
Bread	117.0 (71.7)	121.0 (77.4)	0.761
Sweets	44.6 (37.8)	38.8 (36.7)	0.344
Non-alcoholic beverages	1332.0 (453.0)	1217.0 (547.0)	0.162
Coffee and tea	392.0 (290.0)	386.0 (281.0)	0.863
Alcohol drinks	105.0 (151.0)	121.0 (184.0)	0.686
Animal fat	0.3 (1.0)	0.3 (1.0)	0.782
Vegetable fat	24.9 (17.9)	28.8 (20.2)	0.162
Prepared meals	87.6 (60.8)	76.9 (52.8)	0.162
Salt	0.5 (0.5)	0.4 (0.5)	0.649

Data are shown as mean (SD). [1] Adjusted by energy intake. [2] p was calculated according to the method of Benjamini and Hochberg.

Supplementary Table S2. Daily nutrient intake of the study groups.

Nutrients Intake (units/day) [1]	Normal Glucose Tolerance (n = 319)	Prediabetes (n = 216)	p [2]
Energy intake (Kcal)	2150.0 (544.0)	2183.0 (627.0)	0.986
Glycemic load (%)	98.8 (20.1)	98.1 (24.8)	0.986
Carbohydrate (g)	215.0 (36.4)	212.0 (40.9)	0.986
Carbohydrate (%)	41.7 (6.9)	40.8 (7.3)	
Complex carbohydrate (g)	92.2 (20.8)	93.0 (22.7)	0.986
Complex carbohydrate (%)	17.9 (4.1)	17.9 (4.4)	
Sugar (g)	86.7 (28.1)	82.9 (31.9)	0.986
Sugar (%)	16.9 (5.3)	15.9 (5.1)	
Added sugar (g)	28.5 (18.6)	27.0 (26.5)	0.986
Added sugar (%)	5.7 (3.4)	5.2 (3.9)	
Fiber (g)	22.9 (5.4)	22.6 (5.5)	0.986
Soluble fiber (g)	3.4 (1.0)	3.5 (1.0)	0.986
Insoluble fiber (g)	13.3 (4.5)	13.5 (4.5)	0.986
Protein (g)	97.1 (13.9)	96.4 (17.0)	0.986
Protein (%)	18.7 (2.9)	18.6 (3.4)	
Total fat (g)	89.8 (14.7)	88.5 (16.7)	0.986
Total fat (%)	39.1 (6.5)	39.4 (7.2)	
SFA (g)	26.3 (5.4)	24.9 (5.6)	0.072
SFA (%)	11.4 (2.4)	10.9 (2.3)	
MUFA (g)	42.3 (10.3)	42.6 (11.9)	0.986
MUFA (%)	18.4 (4.5)	19.1 (5.1)	
PUFA (g)	14.3 (3.4)	14.5 (3.7)	0.986
PUFA (%)	6.3 (1.6)	6.5 (1.6)	
Omega 3 (g)	1.5 (0.4)	1.5 (0.6)	0.986
Omega 6 (g)	12.7 (3.3)	12.8 (3.6)	0.986
Trans fat (g)	1.2 (0.5)	1.1 (0.7)	0.986
Cholesterol (mg)	308.0 (87.0)	312.0 (119.0)	0.986
Palmitic acid (16:0) (g)	15.4 (2.6)	14.8 (2.7)	0.267
Stearic acid (18:0) (g)	6.4 (1.5)	5.9 (1.3)	0.011
Oleic acid (18:1ω-9) (g)	39.9 (10.1)	40.3 (11.7)	0.986
Linoleic acid (18:2ω-9) (g)	12.5 (3.3)	12.7 (3.6)	0.986
α-linolenic acid (18:3ω-9) (g)	1.1 (0.2)	1.1 (0.2)	0.986
Arachidonic acid (20:4ω-6) (g)	0.2 (0.0)	0.2 (0.1)	0.986
EPA (20:5ω-3) (g)	0.1 (0.1)	0.1 (0.2)	0.986
DHA (22:6ω-3) (g)	0.3 (0.2)	0.3 (0.3)	0.986
Alcohol (g)	8.3 (13.9)	12.2 (21.3)	0.267
Caffeine (g)	211.0 (222.0)	207.0 (220.0)	0.986
Water (g)	2807.0 (583.0)	2727.0 (706.0)	0.986

Vitamin A (μg)	1160.0 (626.0)	1149.0 (587.0)	0.986
Retinol (μg)	373.0 (398.0)	376.0 (447.0)	0.986
Carotene (μg)	776.0 (437.0)	762.0 (364.0)	0.986
α carotene (μg)	590.0 (569.0)	522.0 (455.0)	0.986
β carotene (μg)	4194.0 (2332.0)	4154.0 (1962.0)	0.986
β cryptoxanthin (μg)	314.0 (198.0)	290.0 (163.0)	0.986
Lutein+zeoxanthin (μg)	3651.0 (2859.0)	3806.0 (2322.0)	0.986
Lycopene (μg)	4061.0 (2360.0)	3769.0 (2067.0)	0.986
Folate (μg)	273.0 (66.9)	271.0 (59.1)	0.986
Vitamin B_{12} (mg)	8.7 (3.6)	8.5 (4.2)	0.986
Vitamin B_6 (mg)	1.9 (0.5)	1.9 (0.5)	0.986
Vitamin C (mg)	111.0 (55.8)	106.0 (45.5)	0.986
Vitamin D (mg)	4.0 (1.5)	3.8 (1.6)	0.986
Vitamin E (mg)	11.3 (3.1)	11.5 (3.5)	0.986
Thiamine (mg)	1.6 (0.3)	1.6 (0.3)	0.986
Riboflavin (mg)	2.2 (0.5)	2.2 (0.5)	0.986
Niacin (mg)	27.0 (5.4)	27.0 (6.5)	0.986
Niacin equivalents (mg)	42.6 (7.0)	42.4 (8.8)	0.986
Calcium (mg)	1072.0 (295.0)	1066.0 (329.0)	0.986
Iron (mg)	13.6 (2.6)	13.4 (2.6)	0.986
Sodium (mg)	3234.0 (527.0)	3212.0 (645.0)	0.986
Potassium (mg)	3259.0 (605.0)	3278.0 (624.0)	0.986
Magnesium (mg)	387.0 (79.3)	388.0 (83.4)	0.986
Zinc (mg)	11.8 (2.0)	11.6 (2.0)	0.986
Selenium (μg)	144.0 (24.1)	143.0 (29.6)	0.986

Data are shown as mean (SD). [1] Adjusted by energy intake. [2] *p* was calculated according to the method of Benjamini and Hochberg. DHA, docosahexaenoic acid; EPA, eicosapentaenoic acid; MUFA, monounsaturated fatty acids; PUFA, polyunsaturated fatty acids; SFA, saturated fatty acids.
